# Homozygous EXOSC3 c.395A>C Variants in Pontocerebellar Hypoplasia Type 1B: A Sibling Pair With Childhood Lethal Presentation and Literature Review

**DOI:** 10.7759/cureus.39226

**Published:** 2023-05-19

**Authors:** Chun Ho Szeto, Sarina Rubin, Richard Sidlow

**Affiliations:** 1 Medical School for International Health, Ben Gurion University of the Negev, Beer Sheva, ISR; 2 Medical Genetics and Metabolism, Valley Children's Hospital, Madera, USA

**Keywords:** pediatric genetics, mendelian, roma, neonatal hypotonia, pediatric rare diseases, spinal muscular atrophy (sma), genotype-phenotype correlations, neurodegenerative disorders, exosc3, pontocerebellar hypoplasia

## Abstract

Pontocerebellar hypoplasia type 1B (PCH1B) is an autosomal recessive neurodegenerative disorder that involves hypoplasia or atrophy of the cerebellum and pons. PCH1B is caused by mutations in *EXOSC3*, which encodes a subunit of the RNA exosome complex. The most frequently observed mutation in PCH1B patients is a c.395A>C (p.D132A) missense variant, for which the homozygous mutation typically results in milder symptoms compared to compound heterozygous mutations or homozygous mutations for other pathogenic variants. In the present study, we report on a sibling pair harboring homozygous *EXOSC3 *c.395A>C missense variants who deteriorated more rapidly than previously described. These cases expand the spectrum of clinical manifestations of PCH1B associated with this variant, highlighting the need for further research to determine predictive factors of PCH1B severity.

## Introduction

Pontocerebellar hypoplasia (PCH) refers to a group of rare heterogeneous neurodegenerative disorders that mainly but not exclusively affect the development of the pons and cerebellum [1]. More than 10 PCH subtypes have been identified based on their clinical and radiologic findings [1]. PCH1 is characterized by anterior horn cell degeneration, resulting in clinical symptoms similar to spinal muscular atrophy (SMA) [1,2]. Patients with PCH1 typically present with muscle weakness, hypotonia, respiratory failure and congenital contractures [1]. The severity of PCH1 varies, depending on the causative genetic mutations and is correlated with individuals' genotypes [3]. Currently, five subtypes of PCH1 have been identified (PCH1A-E) [1].

PCH1B is the most frequently reported subtype of PCH1 associated with the gene *EXOSC3* (OMIM: 606489). *EXOSC3* encodes the non-catalytic subunit of the RNA exosome complex which has a 3’ to 5’ exoribonuclease activity and plays a role in RNA processing and degradation events [4]. Mutations in *EXOSC* severely affect ribosomal RNA (rRNA) processing, and the severity of processing defects has been hypothesized to be correlated with that of the disease course in human patients [4]. However, it remains unknown why cerebellar and motor neurons are particularly susceptible to exosome dysfunctions.

The most prevalent pathologic variant in *EXOSC3* is the c.395A>C (p.D132A) missense mutation [3,5]. This variant alters a highly conserved region which is crucial for RNA binding [6,7]. Patients with the homozygous mutations of this variant often survive into childhood with minimal involvement of the pons, while compound heterozygosity, for instance, with c.155delC, or homozygosity for other *EXOSC3* pathogenic variants has been associated with earlier onset, more profound neurologic findings and shorter lifespans [3,5,6]. *EXOSC3* c.395A>C is not known to be exclusively prevalent in a specific ethnicity, with an allele frequency of 0.0002259 in the Latin/Admixed American population (gnomAD v2.1.1) [3,5]. 

Herein, we report on a sibling pair harboring the homozygous *EXOSC3* c.395A>C missense variant who deteriorated more rapidly than those previously described. Furthermore, we summarize the clinical manifestations of all reported cases with the same mutations.

## Case presentation

Our patients are the third and fourth children of non-consanguineous, healthy parents originating from Mexico. The family history showed no other affected family members.

**Figure 1:**
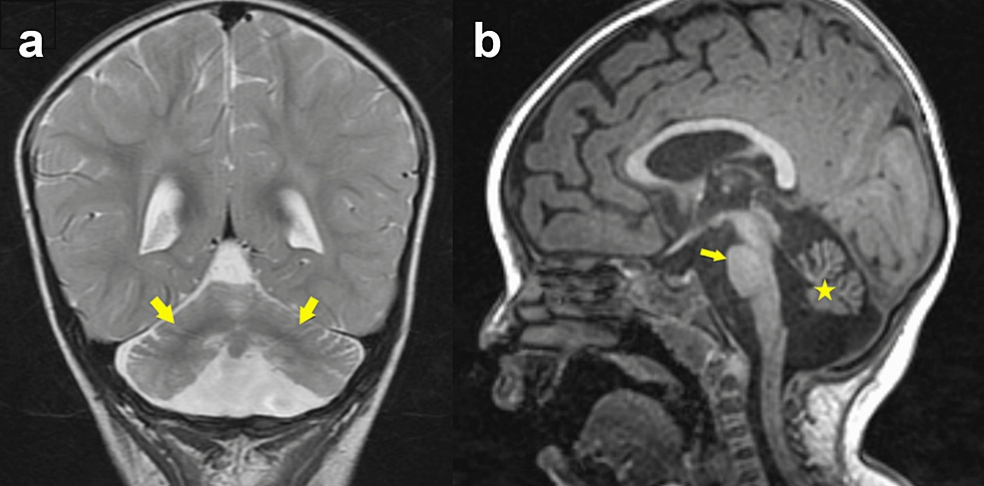
MRI findings in the older sibling. Coronal T2 MRI image (a) demonstrates the splayed hypoplastic appearing bilateral cerebellar hemispheres (arrows). The cerebellum appears to be “floating” in the posterior fossa. Sagittal T1 MRI image in the midline (b) demonstrates a small pons (arrow) and hypoplastic cerebellum (star)

**Figure 2:**
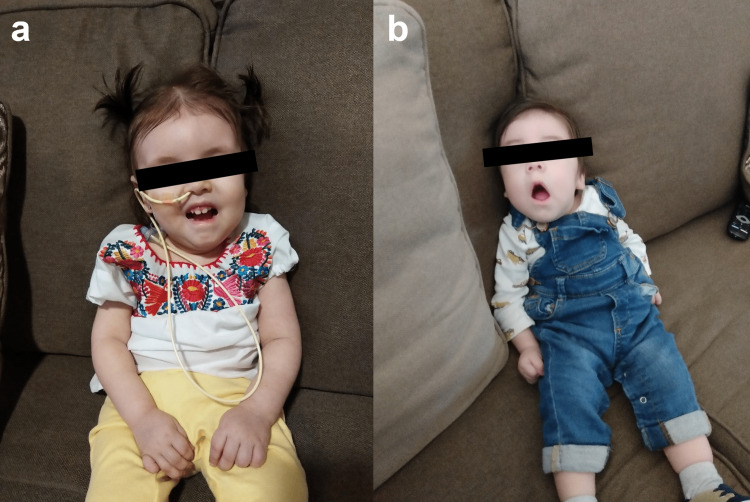
Photographs of (a) the older sibling and (b) the younger sibling. The photos demonstrate myopathic facies and contractures of the hands. Nasogastric tube was inserted on the older sibling for the feeding difficulty.

The older sibling is a female born full-term via normal spontaneous vaginal delivery with no pregnancy or birth complications. Decreased fetal movements were noted during this pregnancy. She presented initially to the Neurology service at one year of age with diffuse hypotonia, weakness, difficulty swallowing and bruxism. Her motor and speech development was delayed based on Denver developmental screening tests. She was not able to roll over or sit, had poor head control, always kept her hands fisted, and was only able to speak two words. Reevaluation at the age of 22 months revealed inability to speak, indicating developmental regression rather than simply delay. An MRI of the brain at this time revealed small bilateral cerebellar hemispheres and bilateral thalami, partial absence of the inferior vermis, and a thinned brainstem, possibly due to atrophy or congenital hypoplasia (Figure 1). There was mild ventriculomegaly and periventricular T2 hyperintensity. Physical examination showed myopathic facies with drooling, generalized sarcopenia, preferential left upward gaze, high arched palate, and contractures of the hands, elbows and knees (Figure 2a).

Initially, the older female sibling was clinically diagnosed with SMA type 1 (SMA1), given her symptom combination and presumptive onset within the first six months of life. However, her findings on MRI were inconsistent with this diagnosis. Based on the above, a Pontocerebellar Hypoplasia Panel (GeneDx, Gaithersburg, MD, USA) was ordered and revealed that she was homozygous for the c.395A>C (p.D132A) variant in the *EXOSC3* gene consistent with the diagnosis of PCH1B. She was treated with 0.5mg Valium twice daily for discomfort caused by bruxism and was offered palliative care treatment. After a number of respiratory illnesses and progressively worsening neurologic status, she died at the age of 28 months. 

The younger male sibling was born three months after the diagnosis of PCH1B was made in his older sister. The pregnancy was equally unremarkable except for decreased fetal movements. Familial variant testing was performed and was positive in this patient as well. Upon examination at the age of two months, he had lower extremity hyperreflexia with spasticity, upper extremity hyporeflexia, myopathic facies with drooling, steeple palate, first toe hypoplasia bilaterally with other toes being long, bilateral foot inversion, low set ears, wandering gaze with left upper gaze preference, long fingers, micrognathia, wide nasal bridge (Figure 2b). He progressively worsened neurologically with multiple hospital admissions for respiratory distress. He was offered palliative care treatment. He died at the age of 19 months.

## Discussion

To date, 22 cases of PCH1B with homozygous *EXOSC3* variants c.395A>C have been reported, including our cases (Table 1) [3,5,6,8-11]. Of these cases, 14 were male and eight were female. At the time of reporting, 13 out of 22 patients (59.1%) had deceased. The median age of reported death was 5.33 years (interquartile range: 3.33-12 years), and our cases were the youngest. The most commonly reported cause of death was respiratory failure (53.8%), followed by other causes including respiratory infection, pseudomonas infection and gastrointestinal failure. Fifteen out of 22 patients (68.2%) survived early childhood (≤6 years). Microcephaly (more than two standard deviations below the average) was present in 63.6% of reported cases. 45.5% and 40.9% of patients were reported to have nystagmus and strabismus, respectively. Feeding difficulties were reported in 11 out of 22 patients (50%), and respiratory failure was present in eight out of 22 patients (36.4%). Hypotonia was present in 95.5% of reported cases, and upper motor neuron signs were present in 54.5% of reported cases. Speech development was absent in 14 out of 22 patients (63.6%) of reported cases. Cerebellar hypoplasia was present in 95.2% of patients on MRI, and variable degrees of pontine hypoplasia were found in 45.5% of reported cases. Salman et al. reported two siblings who presented similarly, but only the sister's exome was found to be homozygous for *EXOSC3* c.395A>C [6,8]. The untested brother who died at the age of 14 months due to severe respiratory failure was not included in our review [8]. Compared to a previous review written by Ivanov et al., our review showed a lower median age of reported death [12].

**Table 1:** Clinical features of 22 reported cases with homozygous EXOSC3 c.395A.C (p.D132A) Abbreviations: CH, cerebellar hypoplasia; F, female; M, male; mo, months; NA, not applicable; NR, not reported; PCH, pontocerebellar hypoplasia; SD, standard deviation; y, years; +, present; -, absent.

Study and Year of Publication	Sex/Origin	Consanguinity	Age (death)	Cause of death	Age at onset	Microcephaly (age: SD)	Motor development	Speech development	Ophthalmologic findings	Neurologic Findings	Respiratory failure	Feeding difficulties	Brain imaging
Our patient (1)	F/Mexico	-	(2.33 y)	Respiratory failure	Birth	-	None	Two words	Strabismus	Diffuse hypotonia, bruxism	+	+	CH, slightly thin brainstem
Our patient (2)	M/Mexico	-	(1.58 y)	Respiratory failure	Birth	-	None	None	Nystagmus	Hypotonia, increased reflexes, hyporeflexia, spasticity	+	-	NR
Nuovo et al., 2020 [11]	F/Italy	NR	9 y	NA	NR	+ (9 y: -6.61)	Partial head control, partial sitting	None	Nystagmus	Proximal muscle hypotonia, hyporeflexia	NR	+	CH, mild pons hypoplasia
Nuovo et al., 2020 [11]	F/Spain	NR	(4.5 y)	NR	NR	- (3 y: -0.37)	None	None	Nystagmus, strabismus	Diffuse muscle hypotonia, hyporeflexia	NR	-	CH, very mild pons hypoplasia
Schottmann et al., 2017 [9]	M/Turkey	+	(15 y)	Respiratory failure	6 mo	+ (6 y: -3.6)	Crawled	None	Nystagmus	Generalized muscular hypotonia, areflexia, increased reflexes	+	+	CH
Eggens et al., 2014 [5]	F/Western Eurasian ancestry	NR	(7 y)	Respiratory failure	Birth	- (4.5 mo: +3)	NR	babble	Nystagmus	Hypotonia, increased reflexes, dykinesia	+	+	CH
Eggens et al., 2014 [5]	M/Western Eurasian ancestry	NR	(12 y)	GI failure	Birth	- (11 y: -0.5)	NR	Single words	Nystagmus	Hypotonia, increased reflexes, dyskinesia, seizures	NR	+	CH
Eggens et al., 2014 [5]	M/Western Eurasian ancestry	NR	(10 y)	Pseudomonas infection	Birth	+ (6.5 y: -2)	NR	Single words	Nystagmus	Hypotonia, increased reflexes, dyskinesia	NR	+	CH
Rudnik-Schöneborn et al., 2013 [3]	F/Turkey	+	20 y	NA	3 mo	+ (15 y: -3.5)	Head control, sitting, crawled, few steps with support	Single words	Strabismus	Hypotonia, increased reflexes, dystonia, spasticity, weakness	-	+	PCH
Rudnik-Schöneborn et al., 2013 [3]	M/Turkey	+	16 y	NA	3 mo	+ (2 y: -2)	Head control	None	Strabismus	Trunk hypotonia, increased reflexes, myoclonus, spasticity	-	-	PCH
Rudnik-Schöneborn et al., 2013 [3]	F/Germany	-	18 y	NA	<6mo	- (18 y: 0)	Abduction pant until 6 mo (hip dysplasia)	None	Nystagmus, strabismus	Hypotonia, myoclonus, absence epilepsy	+	+	CH
Rudnik-Schöneborn et al., 2013 [3]	F/Germany	-	(5.33 y)	NR	3-6 mo	- (4 y: -0.5)	Head control, turned around	None	Nystagmus, strabismus	Hypotonia	-	+	PCH
Biancheri et al., 2013 [10]	M/Italy	-	14 y	NA	1 mo	+	Supported walking	None	Strabismus	Diffuse muscle hypotonia, increased reflexes, Babinski sign	NR	+	CH
Biancheri et al., 2013 [10]	M/Italy	-	7 y	NA	1 mo	+	Unsupported sitting	None	Strabismus	Axial muscle hypotonia, increased hyperreflexia, limbs hypertonus, Babinski sign	NR	NR	CH
Biancheri et al., 2013 [10]	M/Italy	+	(5 y)	Acute cardiorespiratory failure	2 mo	+	Supported sitting	None	NR	Axial muscle hypotonia, limbs hypertonus, Babinski sign	+	NR	CH
Biancheri et al., 2013 [10]	M/Italy	+	(14 y)	Acute cardiorespiratory failure	<6 mo	+	Partial head control	Single word	Strabismus	Axial muscle hypotonia, limbs hypertonus, Babinski sign	+	NR	CH
Wan et al., 2012 [6]	M/America and Europe	-	(18 y)	Respiratory infection	Birth	+	None	None	Ocular apraxia	Hypotonia	NR	NR	CH, brainstem normal configurations but small
Wan et al., 2012 [6]	M/America and Europe	-	18 y	NA	Birth	+	None	None	Ocular apraxia	Hypotonia	NR	NR	CH, brainstem normal configurations but small
Wan et al., 2012 [6]	M/America and Europe	-	16 y	NA	Birth	+	None	None	Ocular apraxia	Hypotonia	NR	NR	CH, brainstem normal configurations but small
Wan et al., 2012 [6]	M/America and Europe	-	9 y	NA	Birth	+	None	None	Ocular apraxia	Hypotonia	NR	NR	CH, brainstem normal configurations but small
Wan et al., 2012 [6]	M/ Australia and Turkey	+	(3 y)	NR	NR	NR	NR	NR	NR	NR	NR	NR	NR
Salman et al., 2002; Wan et al., 2012 [6,8]	F/Canada and Cuba	-	(3.33 y)	Respiratory failure	5 mo	+	Supported sitting	NR	Nystagmus, retinal dystrophy	Diffuse muscle hypotonia, increased reflexes, limbs hypertonus, areflexia	+	+	PCH

Phenotypes for PCH1B can be categorized into three subgroups: mild, moderate and severe [13]. Severe phenotypes are associated with certain *EXOSC3* genotypes; for instance, patients with homozygous c.92G>C consistently die during infancy [5,12]. Patients with mild PCH1 manifest with normal appearance at birth, psychomotor retardation within the first six months of life, preserved respiratory function and a prolonged disease course with survival beyond infancy [3,10,13]. So far, the mildest form of PCH1B was reported by Mu et al., whose cases were compound heterozygous for *EXOSC3* c.155delC and c.80T>G [14]. Homozygosity for *EXOSC3* c.395A>C has been proposed to lead to mild PCH1, but as shown in our review, around 30% of patients presented with moderate PCH1 and died during early childhood. Our patients had the earliest age of death among all reported cases with homozygous *EXOSC3* c.395A>C mutations, and they are the first documented cases with such a genotype who died below the age of three years.

While genotype-phenotype correlations have been established, it is still unclear why patients with the same *EXOSC3* genotype exhibit different severity of phenotypic expression [3,5]. The causes could be multifactorial, involving the interplay of other genes, epigenetics and environmental factors. Le Duc et al. suggested that male sex might be positively associated with PCH1 disease severity [15]. However, our review appears to disagree with this speculation. Compared to female sex, male sex is not statistically significantly associated with death by the age of six years (OR: 0.273, CI: 0.041 to 1.795). This calculation of odds ratio is apparently subject to selection bias, as seen in many speculations drawn in case studies. Therefore, a large cohort of PCH1 patients is needed to identify predictive factors associated with disease severity. 

The initial presentations of SMA1 and PCH1 overlap in the following ways: they initially present with severe weakness before six months of age, along with challenges with breathing, coughing, and swallowing [16]. Their courses progress similarly in that there is difficulty feeding, delayed motor milestones, and an increased risk of death via aspiration pneumonia due to progressed muscle weakness [17]. Spinal MRI for both entities can show anterior horn degeneration and both diseases can cause severe and diffuse sarcopenia [17,18]. Given these similarities, it can be difficult to differentiate between SMA1 and PCH early in life. However, it is imperative to distinguish between the two conditions in the neonatal period given the availability of medications for SMA1. Nusinersen, for example, is one of three FDA-approved medications that can significantly improve motor functions and event-free survival in SMA patients [19]. In contrast to SMA, PCH is currently untreatable and patients are invariably offered palliative care or a combination of physical therapy, ventilation support, anti-seizure medications and tube feeding, if necessary [3]. Given the difficulty in differentiating PCH1B from SMA1 based solely on clinical symptoms, the currently available prenatal screening tests for SMA1 are timely. In addition, researchers have recommended PCH1B screening for couples of Romani origin, specifically targeting the *EXOSC3* c.92G>C variant, as this rare pathogenic variant was mainly reported in the Romani population [5,20].

## Conclusions

Our case report and literature review refine the clinical spectrum of the most common subtype of PCH1B. Given the broad spectrum of clinical manifestations, differentiating between PCH1B from SMA can be challenging due to the overlapping presentations. The underlying reasons for phenotypic variation despite the same* EXOSC3* genotype remain unclear. Therefore, further research is required to identify predictive factors of severity and elucidate the pathomechanism of PCH1B. 
